# Breath-Focused Mindfulness and Compassion Training in Parent-Child Dyads: Pilot Intervention Study

**DOI:** 10.2196/69607

**Published:** 2025-07-17

**Authors:** Satish Jaiswal, Jason Nan, Seth Dizon, Jessica O Young, Suzanna R Purpura, James K Manchanda, Dhakshin Ramanathan, Dennis J Kuo, Jyoti Mishra

**Affiliations:** 1 Department of Psychiatry, University of California, San Diego, 9500 Gilman Drive, Guava Building, Room 130, La Jolla, CA, 92093, United States, 1 6086092291; 2 VA San Diego Medical Center, San Diego, CA, United States

**Keywords:** child, parent, depression, compassion, mindfulness, default mode network, EEG, electroencephalograph

## Abstract

**Background:**

Depression in children is a concerning societal issue and can be associated with poor academic performance, school dropout, and poor overall quality of life. Additionally, child depression is often associated with parallel stress and depression in parents.

**Objective:**

This scenario highlights the urgent need for the development and implementation of accessible and scalable solutions that may cobenefit child and parent mental well-being.

**Methods:**

This pilot study introduced “Cooperative Compassion” (CoCo), a parent-child cotraining digital application aimed at promoting mindfulness and compassion through brief, performance-adaptive sessions. A community sample of 24 parent-child dyads (children’s mean age 9.5, SD 3.27 years; female: n=14, male: n=10; Asian: n=5, White: n=11, mixed race: n=7, other race: n=1; and parents’ mean age 44.5, SD 6.5 years; 20 female: n=20, male: n=4; Asian: n=8, White: n=14, mixed race: n=2) of high average affluence socioeconomic scores participated in the study. These parent-child dyads completed 30 sessions of CoCo training over 3 months with baseline and postintervention assessments occurring within 2 weeks of training initiation or completion, respectively.

**Results:**

The program was feasible, with 80% (n=19) of families completing over 90% (n=22) of sessions and providing positive feedback. Mental health assessments showed a nonsignificant effect in the expected direction in children’s depression scores (Cohen *d*=−0.19; 95% CI −8.89 to 1.74; *P*=.07) and significant reductions in parental stress (*d*=−0.41; 95% CI −2.63 to −0.16; *P*=.02), anxiety (*d*=−0.47; 95% CI −2.67 to −0.20; *P*=.02), and depression (*d*=−0.50; 95% CI −3.25 to −0.08; *P*=.03), with sustained benefits at the 3-month follow-up. Parental mindfulness improvements were correlated with stress reduction (ρ=−0.45; *P*=.03). On an emotion bias task used as an objective assessment of cognition, children demonstrated improved processing speed after the intervention (*d*=0.54; 95% CI 0.012-0.083; *P*=.005), and a marginal improvement was also observed in parents (*d*=0.19; 95% CI −0.004 to 0.030; *P*=.05). Cortical source imaging of electroencephalographic recordings was acquired simultaneous to an attention-to-breathing assessment that showed significant reduction in task-related default mode network activity (*d*=−0.62; 95% CI −0.0096 to −0.0002; *P*=.01).

**Conclusions:**

Post-CoCo intervention decrease in default mode network activity on the attention-to-breath task in parent-child dyads may be indicative of cortical plasticity reflecting reduced mind-wandering and thereby, enhanced focus after training. The current promising behavioral and cognitive results suggest the need for a larger sample size and a randomized controlled study design. Overall, these findings highlight the potential for brief, digital mindfulness and compassion cotraining to improve family mental health and well-being.

## Introduction

The Centers for Disease Control and Prevention estimates a 5% prevalence of depression in US children [1]. Depression in children is comorbid with anxiety and attention deficit [2-5] and may be associated with poor academic performance and school dropout, foretelling poor overall quality of life for these children [6,7]. In serious cases, children with depression can be susceptible to the grave risk of suicide [8]. Experts note that this scenario has been further exacerbated by the COVID-19 pandemic [9-11]. Additionally, depression in children is often associated with parental stress and concomitant parental depression [12-14]. Overall, this scenario highlights the urgent need for the development and implementation of accessible and scalable solutions that cobenefit child and parent mental well-being.

Parents play significant roles as either supportive influences, protective buffers, or potential sources of risk depending on the nature of their caregiving relationship with their children [15,16]. Several studies have underscored the detrimental mental health effects on children of poor parental mental health and psychological distress [17-19]. Additionally, developmental studies on parent-child dyads have consistently shown that parental involvement not only affects child behaviors but also influences emotion regulation as well as cognitive and neural responses in children [20-24]. With regard to interventions, a recent meta-analytic study [25] of parent-child mental health interventions recommended that interventions should recognize the family as an integrated unit by taking into account the collective needs of parents and children. By offering external guidance and emotional scaffolding, caregivers help shape children’s ability to regulate their emotions—a process referred to as coregulation [26,27]. Thus, we hypothesize that parent-child cointervention programs that promote joint participation may leverage coregulation to foster parent-child co–well-being. Of note, research also shows that in this context, families prefer nonpharmacological treatments for their children whenever effective behavioral solutions are available [28].

To address this intervention gap, we developed and implemented a digital parent-child cotraining program that focuses on imparting mindfulness and compassion skills to both parent and child. Our focus on designing a digital program was driven by the need for greater accessibility and scalability of such interventions. Studies have shown that mindfulness and compassion training in children and parents can benefit interpersonal behaviors and even improve stress physiology [29-32]. For instance, a study of mindful parenting training found significantly reduced posttraining psychopathology in parents as well as in their children [33]. Another study demonstrated that Cognitively-Based Compassion Training in parents significantly reduced stress cortisol levels in their infants and young children [31]. Furthermore, many school-based mindfulness interventions have been introduced to enhance well-being in children, and such programs have grown rapidly since the pandemic [30,34,35]. Yet, all of these studies have examined the efficacy of mindfulness or compassion-based intervention separately applied to parents or children but not as cotraining. A recent randomized controlled trial compared parallel (but not simultaneous) parent and child mindfulness training to pharmacological intervention in children with attention-deficit/hyperactivity disorder (ADHD) and concluded that mindfulness training can be considered a useful nonpharmacological alternative or add-on to pharmacological treatment [36]. Other studies have also shown the utility of parent and child parallel mindfulness trainings in ADHD [37,38]. In addition, in a previous study, we showed that digitally delivered meditative trainings can benefit attentive function in adolescents who report childhood neglect and show signs of ADHD [39]. Overall, this literature suggests that a parent-child mindfulness and compassion cotraining application is an innovative approach.

In the interventional program that we designed and implemented, parent-child dyads jointly participated in working through 10-minute attention-to-breath mindfulness exercises in each session alongside prompts for compassion cultivation. Each session was kept brief to facilitate adherence in busy families, and up to 30 training sessions were available for families to practice over 3 months. We referred to the program as Cooperative Compassion (CoCo) training, and both parent and child underwent the practices together. To evaluate the outcomes of this intervention, we used self-report mental health measures at baseline, after training, and 3-month follow-up and also measured objective cognition in the presence of emotions at post- versus pretraining [40-43]. Additionally, we acquired electroencephalographic (EEG) recordings on an attention-to-breath assessment at pre- and posttraining as a neural outcome measure in all participants [44,45].

Since prior intervention research in this field has mostly relied on self-report measures and there have been limited comprehensive attempts at examining the effects of longitudinal parent-child cointervention programs [25,46-48], in this pilot study, we used multimodal assessments, that is, a combination of self-report mental health measures, an objective emotion-based cognitive processing measure, and a neural EEG-based measure in both parents and children. We hypothesized that after CoCo training, we would observe an improvement in mental health behaviors as the primary outcome and changes in cognitive processing and underlying neuroplasticity in both children and parents as secondary outcomes. Additionally, in the context of emotion regulation, prior interventions have focused on changing and evaluating parental behaviors but with limited assessment of children’s emotion processing skills [22]. Therefore, in this study, we explored the dynamics of emotion-related cognitive processing changes in both children and parents. Finally, we used an EEG-based neural recording on an attention-to-breath assessment, using variability in breath-monitoring responses as a direct objective marker of variability in performance on an interoceptive attention task [45]. We adopted this task, as we have found that neurophysiology on this task correlates with trait mindfulness in a large cross-sectional study in individuals across the lifespan and have further demonstrated that this neurophysiology undergoes plasticity in the context of a mindfulness and compassion intervention in adults [44]. Specifically, we have observed intervention-driven reduction in default mode network (DMN) activity on the attention-to-breathing task demonstrating posttraining plasticity of the DMN. Using neuroimaging, several other studies have shown that DMN activity is modulated by contemplative interventions [49-52]. The DMN is a functional network that has been consistently associated with autobiographical memory, self-referencing, but also with on-task behavioral variability, mind-wandering, and rumination [53-58]. Given its role in mind-wandering, we specifically hypothesized that the CoCo training would drive a reduction in DMN activity at postintervention relative to baseline. In summary, we hypothesized improvement in mental health behaviors as the primary outcome and improvement in emotion-related cognitive processing and reduction in EEG-derived DMN activity, reflective of reduction in mind-wandering and enhancement of present moment focus, as secondary outcomes.

## Methods

### Participants

A total of 24 parent-child dyads participated in this study. Dyads were recruited from the local community, that is, schools and university-affiliated pediatric clinics in the San Diego area through flyer advertisements and clinic referrals, respectively. The recruitment of parent-child dyads began on February 1, 2021, and ended on August 13, 2023. The study was registered on October 9, 2024, in the International Standard Randomized Controlled Trial Number Registry (ISRCTN89594822).

### Ethical Considerations

Signed informed consent was obtained from the parents, and signed assent was obtained from the children for study participation following the guidelines outlined in the Declaration of Helsinki. The study protocol was approved by the institutional review board of the University of California San Diego (protocol #180140). The study data were deidentified as per Health Insurance Portability and Accountability Act (HIPAA) compliance. All the parent-child dyads were paid US $250 for completing all assessments and digital trainings.

### Demographics

Parent and child demographics including age, sex, race, ethnicity, and socioeconomic status (SES) scores are shown in Table 1. SES was measured on the Family Affluence Scale [40,59-64]; this scale measures individual wealth based on ownership of objects of value (eg, car or computer) and number of vacations in the past year. The sum of items produces a composite score ranging from 0‐2=low affluence, 3‐5=middle affluence, and >5=high affluence. The average family SES score in our study sample was of high affluence. All children and parents were right-handed and had normal or corrected-to-normal vision.

**Table 1.** Summary of demographics and baseline mental health for parent-child dyad study participants^a^.

Demographics and baseline mental health	Parents (n=24)	Children (n=24)
Age (years)		
Mean (SD)	44.5 (6.5)	9.5 (3.27)
Range	5‐12	28‐54
Sex, n (%)		
Male	4 (16.7)	10 (41.7)
Female	20 (83.3)	14 (58.3)
Race, n (%)		
Asian	8 (33.3)	5 (20.8)
Black or African American	0 (0)	0 (0)
Native American	0 (0)	0 (0)
Native Hawaiian or Other Pacific	0 (0)	0 (0)
White	14 (58.3)	11 (45.8)
More than 1 ethnicity	2 (8.3)	7 (29.2)
Other	0 (0)	1 (4.2)
Ethnicity, n (%)		
Hispanic or Latino	5 (20.8)	5 (20.8)
Not Hispanic or Latino	18 (75)	19 (79.2)
Unknown	1 (4.2)	0 (0)
Socioeconomic status		
Mean (SD)	6.5 (1.4)	—^b^
Range	4-8	—
Child Depression Index *T* scores		
Mean (SD)	54.08 (9.06)	57.96 (13.75)
Range	40-90	37-74
Parental stress (DASS-21)		
Mean (SD)	4.91 (3.61)	—
Range	0‐13	—
Parental anxiety (GAD-7)		
Mean (SD)	4.39 (3.28)	—
Range	0-14	—
Parental depression (PHQ-9)		
Mean (SD)	5.13 (3.52)	—
Range	0‐13	—

a Parental stress was measured using the 7 stress items on the 21-item Depression Anxiety Stress Scale (DASS-21), anxiety was measured on the 7-item General Anxiety Disorder (GAD-7) scale, and depression was measured on the 9-item Patient Health Questionnaire scale (PHQ-9).

b Not available.

Child and parent baseline mental health scores are also shown in Table 1. In our community-recruited sample, children were assessed on the Child Depression Index (CDI) [40,59-65] for study inclusion, that is, must have average or above-average CDI scores (*T* score>40). Parents in the study did not report any diagnosed illness. Exclusion was based on any self-reported severe illness for parent or child that would not allow time for study participation.

### Sample Size and Power

This single-arm study was powered to detect a medium effect size (Cohen *d* >0.5) comparing pre- versus postintervention differences at β power of .8 and α level of .05 for each assessment measure (behavioral, cognitive, and neural). Effect sizes were calculated a priori using the G*Power software (Heinrich Heine Universität) [66].

### Feasibility

We assessed intervention feasibility by monitoring the total number of assigned intervention sessions completed. Additionally, parents completed a feasibility survey at the end of the study that we have standardized in previous digital training studies [67]. The survey queried 16 questions about the training as elaborated in the Results section (Table 2), and each question required a response on a 7-point Likert scale. In total, 11 of 16 questions solicited positive feedback regarding the intervention, while 5 questions solicited negative feedback and are shown in italics format in Table 2; for all questions, anchors were 1=strongly disagree to 7=strongly agree, and negative feedback questions were reverse coded and then averaged with positive questions to obtain overall training feasibility. The Cronbach α measure of reliability for the training survey was high (α=0.92).

**Table 2.** Results of the training feasibility survey completed by all participating parents (N=24) at the end of the study.

Training feasibility survey	Response on 1‐7 scale, mean (SD)
We enjoyed the training.	4.96 (1.76)
*We felt frustrated after the training*.^a^	3.33 (2.18)
We felt satisfied with the training.	4.88 (1.85)
*We felt tired after the training*.	3.17 (1.74)
The training was easy to understand.	5.67 (1.71)
*The training was difficult to use*.	2.63 (1.69)
The training was easy to navigate.	5.75 (1.07)
*We were worried about our data security*.	1.58 (0.88)
The training was easy to initiate each day.	5.29 (1.71)
The training fit in our daily schedule.	4.79 (1.86)
The training time passed by quickly.	4.13 (1.83)
The training felt therapeutic.	4.21 (1.93)
*The training felt useless*.	3.21 (1.93)
We would recommend this training outside of this study.	3.96 (2.26)
We would recommend this training to others.	4.08 (2.28)
This training positively affected our family’s life.	4.25 (1.85)
Average positive feedback	4.72 (1.83)
Average negative feedback	2.78 (1.68)
Average program feedback	4.97 (1.76)

a Questions soliciting negative feedback are marked in italics format.

### Assessments

#### Overview

**Figure 1.**
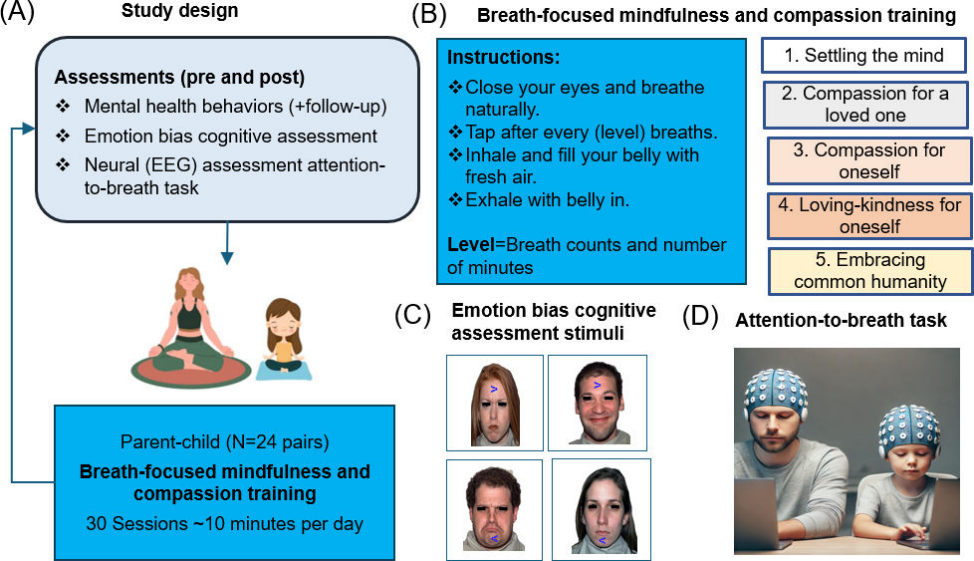
Study design and breath-focused compassion training. (**A**) The study design incorporated behavioral, cognitive, and EEG assessments at pre- and posttraining conducted in parent-child dyads. Behavioral assessments were additionally repeated at a 3-month follow-up after intervention completion. (**B**) The digital *Cooperative Compassion* training delivered attention to breath-focused, performance-adaptive training in which the parent and child engaged simultaneously. {Level} refers to the number of breaths monitored by both parent and child ranging from 1 to 10. Higher levels were accessed when performance at lower levels was consistent for at least two-thirds of the level time. Performance was monitored, as users tapped the mobile screen after instructed {level} number of breaths while keeping their eyes closed; the screen was digitally split to simultaneously track both parent and child performance. At every sixth session of training, compassion instructions were relayed by text and audio before the start of the breath-focused exercises so that users could discuss and keep these instructions in mind during their practice. There were a total of 5 levels of standard compassion training instructions provided focusing on settling the mind, compassion for a loved one, compassion for self, loving kindness for self, and embracing common humanity. A distinct, calming nature scene unveiled at the end of each session. (**C**) Stimuli for the emotion bias cognitive assessment are shown and presented neutral, happy, sad, or angry faces superimposed on an arrow, whose direction was discriminated by participants. (**D**) In the attention-to-breath task, participants were instructed to close their eyes, breathe naturally, and respond after every 2 breaths by tapping on the spacebar, while simultaneously EEG was being recorded. EEG: electroencephalography.

Each parent-child dyad made 2 visits (baseline [preintervention] and 3 months later [postintervention]) to the Neural Engineering and Translational Labs and participated in behavioral and neurocognitive assessments (Figure 1A); these assessments were conducted within 1‐2 weeks of intervention initiation or completion, respectively. Behavioral assessments were also completed on the web at a 3-month follow-up after intervention completion.

#### Behavioral Assessments

Each child self-reported on the CDI, and parents also provided CDI reports for their child. The CDI self-report and parent report are 12-item and 17-item scales, respectively, with higher scores reflecting greater child depression. Each CDI child report question requests the child to pick 1 of 3 sentences based on which one best described their emotion or behavior over the past 2 weeks rated 0=least to 2=most negative emotion or behavior. The CDI parent report enquires about child emotions or behaviors on a 4-point scale with anchors 0=not at all to 3=much or most of the time. Each parent additionally completed standard surveys assessing their own mental health including stress, anxiety, and depression, evaluated using the 21-item Depression Anxiety Stress Scale (DASS-21) with 4-point anchors from 0=did not apply to me at all to 3=applied to me very much or most of the time [68], 7-item General Anxiety Disorder (GAD-7) scale with 4-point anchors from 0=not at all to 3=nearly every day [69], and the 9-item Patient Health Questionnaire (PHQ-9) for depression scale with same anchors as for GAD-7; higher scores in each of these scales denoted greater symptom severity [70]. Parents also completed a 14-item mindfulness measure, Mindful Attention Awareness Scale (MAAS), that inquires about being unaware in the present moment in the context of different daily life activities with 6-point anchors from 1=almost always to 6=almost never, and higher scores reflecting higher trait mindfulness [71]. The Cronbach α measure of reliability calculated at baseline was high for each of these measures (CDI child report: α=0.83, CDI parent report: α=0.83, DASS-21: α=0.89, GAD-7: α=0.84, PHQ-9: α=0.74, MAAS: α=0.94).

#### Cognitive Assessment

Participants accessed a game-like emotion bias assessment adapted from studies of attentional bias in emotional contexts [40-42,71]. The task was delivered on the BrainE Unity-coded platform developed and deployed by Neural Engineering and Translational Labs [60,72] with stimuli displayed at a comfortable viewing distance. The task integrated a standardized set of culturally diverse faces from the NimStim database [73], examples shown in Figure 1C. We used an equivalent number of male and female faces, each face with 4 sets of emotions: neutral, positive (happy), negative (sad), or threatening (angry), presented on an equivalent number of trials in each task block. Postfixation cue on each trial, participants viewed an emotional face with a superimposed arrow of 300-millisecond duration. The arrow occurred in either the upper or lower central visual field on an equal number of trials. Participants responded to the direction of the arrow (left or right) within an ensuing 1-second response window. For neutral emotion trials, this response window adapted in a 3-up-1-down scheme (−33 ms after correct trials and +100 ms after incorrect trials) that maintains accuracy at ~80% and engages the user by avoiding ceiling performance [67,74]. An adaptive scheme also reduces practice effects that affect repeat assessment sessions. All other emotion trials followed the same response window as their previous neutral emotion trial. Participants completed 144 trials presented over 3 equipartitioned blocks. Processing speed across trials was monitored as the main outcome of this assessment.

#### Neural (EEG) Assessment

We assessed EEG neurophysiology on an attention-to-breathing task assessment that we have recently shown to relate to mindfulness in a large cross-sectional study [45] and further show modulation in the context of a mindfulness and compassion intervention in adults [44]. As described in these studies, participants were instructed to close their eyes, breathe naturally, and respond every 2 breaths by tapping on the spacebar. The lab streaming layer protocol was used to time-stamp all user response events. The task was of 5-minute duration, implemented in two 2.5-minute blocks.

The median response time (RT) on the breath monitoring task was monitored for all participants, so that we could identify and contrast neurophysiological activity on high consistency breath monitoring trials (trials with RT ≤1 median absolute deviation of median RT) versus low consistency trials (trials with RT >1 median absolute deviation of median RT). The underlying premise of this analysis is that when participants are in a state of higher interoceptive attention, they will show more consistent awareness of their respiratory cycle (ie, responding more consistently after every 2 breath cycle), while states of lower interoceptive attention may be associated with inconsistent breath monitoring. Thus, by comparing brain activity on more versus less consistent trials within each participant, we can examine brain activity underlying performance variability on this interoceptive attention task while controlling for interindividual differences in baseline characteristics of respiration.

EEG data were collected simultaneous to this assessment using a 24-channel cap with saline-soaked electrodes following the 10‐20 system and a wireless SMARTING amplifier. The signals were digitized with a sampling rate of 500 Hz and 24-bit resolution and stored as .xdf files.

### Intervention

Akin to a similar intervention that we deployed in adults [44], the digital CoCo training for parent-child dyads was delivered on the HIPAA-compliant BrainE app with a deidentified and password-protected login provided to each family. Participants accessed the iOS- or Android-compatible smartphone app in their own free time and engaged in ~10 minutes of training per session for up to 30 sessions (Figure 1B). The training was delivered in a game-like format and was performance-adaptive. The single-user interface design was already vetted in our prior study in adults [44]. For this study, the dual user interface for parent-child dyads was informally tested for usability by the study team’s family members.

Specifically to practice breath-focused mindfulness, individuals were requested to close their eyes, pay attention to their breathing, and tap the mobile screen after a specific number of breaths from 1 breath up to 10 breaths at a time. Consistency of performance was monitored, as users tapped the mobile screen after instructed number of breaths while keeping their eyes closed; the screen was digitally split to simultaneously track both parent and child performance, that is, one-half of the screen kept track of child finger taps, while the other half kept track of parent taps. If both parent and child consistently monitored breathing for at least two-thirds of the duration at any given level, where level refers to the number of breaths monitored, then they would together progress to monitoring the next level or count of breaths. At every sixth session of training, compassion instructions were relayed by text and audio before the start of the breath-focused exercises so that users could discuss and keep these instructions in mind during their practice. Over 30 sessions, there were a total of five levels of standard compassion training instructions provided focusing on (1) settling the mind, (2) compassion for a loved one, (3) compassion for self, (4) loving kindness for self, and (5) embracing common humanity; these instructions followed guidance from the Compassion Cultivation Training program [75]. These compassion training instructions appeared as text and audio on the app’s daily introductory screen; the text details at each level have been provided in Multimedia Appendix 1. Finally, a distinct, calming nature scene unveiled at the end of each session as a form of training reward. Parents received app notifications once a day reminding them to complete their training.

### Data Analyses

Behavioral outcomes were analyzed using paired tests comparing pre- and posttraining assessments as well as pre- and follow-up assessments. Outliers >3 median absolute deviation from the median were removed, and all metrics were inspected using the Anderson-Darling normality test in MATLAB prior to statistical analyses. All measures, that is, CDI reports from children and parents and parental stress (per DASS-21), anxiety (GAD-7), and depression (PHQ-9) measures, were not normally distributed; hence, they were analyzed for pre- to posttraining changes using signed rank tests. Parent mindfulness data were normally distributed and hence, analyzed using 1-tailed *t* tests. One-sided paired tests were used in all cases, as score improvement in a single direction was meaningful for all measures. While 2-sided tests are more conservative, we have ensured that only strong effects are reported by applying (fdr) corrections for multiple statistical comparisons.

Cognitive outcomes corresponded to the pre- to postintervention difference in processing speed on the emotion bias task. These data were not normally distributed, and pre- to posttraining differences in processing speed across participants were analyzed using the signed rank test, with group as a factor analyzed using the Wilcoxon rank sum test.

Neural data on the eyes-closed attention-to-breathing task were analyzed according to methodologies used in our recently published studies [44,45]. Adult parent data were available for 22 of 24 parents, and child data were available for 19 of 24 children; thus, overall source analysis was conducted on 41 samples with children and adults data combined with individual age-specific head models applied (Multimedia Appendix 1) [76-78]. EEG data from the remaining adults or children were too noisy for analysis and were excluded; no imputations were made for these missing data. EEG analysis included (1) EEG channel data processing and (2) cortical source localization of the EEG data to estimate source-level neural activity. Details of this analysis are provided in Multimedia Appendix 1. Briefly, cleaned EEG data were epoched to the response taps made by participants after every 2 breaths with trials separated by either high consistency (trials with RT ≤1 median absolute deviation of median RT in each participant) or low consistency (trials with RT >1 median absolute deviation of median RT in each participant). As α band (8‐12 Hz) oscillations are dominant in the eyes-closed state, source localized activity was analyzed in the α band in 3 canonical brain networks, the frontoparietal network (FPN), the cingulo-opercular network (CON), and the DMN. Specifically, we quantified the average network source activity for the low versus high consistency trial differential in the 0‐ to 4-second period prior to breath response, as this provided a neural correlate for low versus high consistency task performance. Neural data were not normally distributed as verified by the Anderson-Darling test; hence, the pre- to posttraining difference in α source activity across participants was analyzed using the signed rank test in each of the 3 networks (FPN, DMN, and CON), with group as a factor analyzed using the Wilcoxon rank sum test.

## Results

### Intervention Feasibility

On average, families completed a mean of 27.58 (SD 4.82) of the total 30 assigned sessions, with 19 of 24 families completing more than 90% of the assigned sessions.

All parents also completed a feasibility survey (Table 2) responding to feasibility questions on a 7-point Likert scale. The average positive feedback across questions such as “We enjoyed the training,” “We felt satisfied with the training,” “The training was easy to understand,” and “This training positively affected our family’s life” was 4.72 (SD 1.83). In contrast, the average negative feedback across questions such as “We felt frustrated after the training,” “We felt tired after the training,” “The training was difficult to use,” and “The training felt useless” was 2.78 (SD 1.68). Overall, training program feedback was positive, that is, a mean response score of 4.97 (SD 1.76) of 7.

### Behavioral Outcomes

Pre- versus postintervention change in CDI scores for children by self-report showed a nonsignificant effect in the expected direction (signed rank test *z*=1.50; Cohen *d*=−0.19; 95% CI −8.89 to 1.74; *P*=.07). Change in parent-reported CDI scores was nonsignificant (*z*=1.05; *d*=−0.20; 95% CI −4.93 to 1.27; *P*=.15). Parental stress (DASS-21, *z*=2.03; *d*=−0.41; 95% CI −2.63 to −0.16; *P*=.02), anxiety (GAD-7, *z*=2.15; *d*=−0.47; 95% CI −2.67 to −0.20; *P*=.02), and depression (PHQ-9, *z*=1.96; *d*=−0.50; 95% CI −3.25 to −0.08; *P*=.03) were all significantly improved at the posttraining time point.

**Figure 2.**
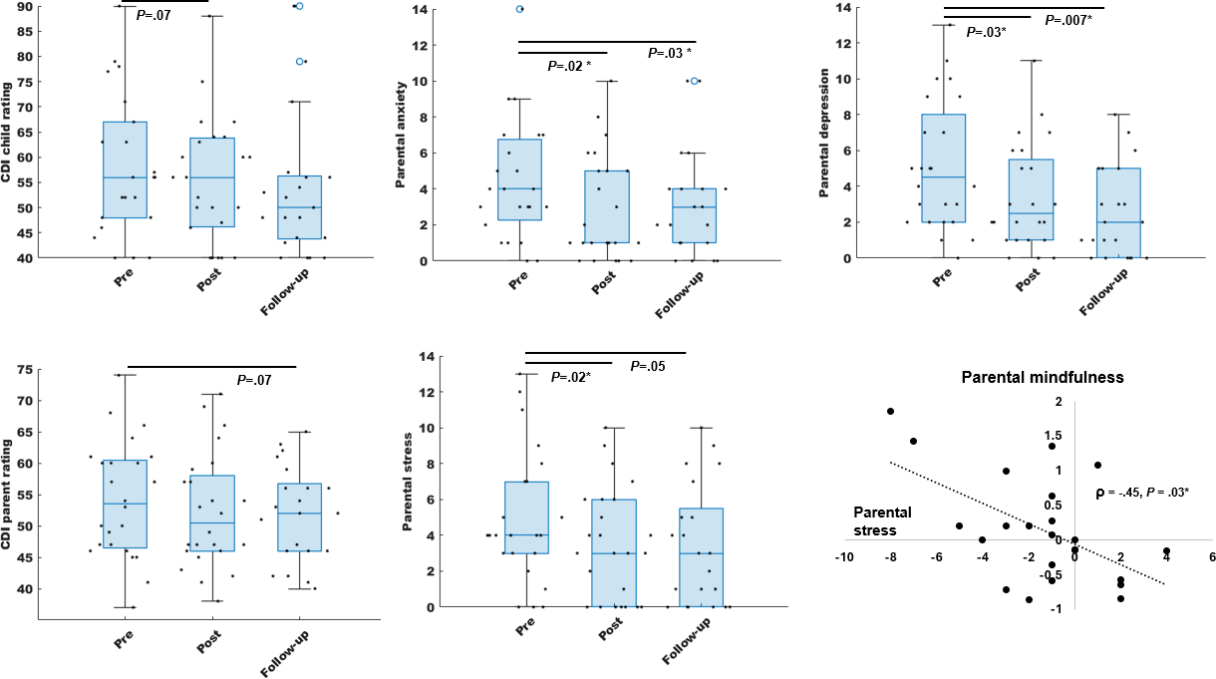
Swarm box charts of behavioral outcomes are shown for children (left column) and parents (middle and right columns). Box plots show the median with the lower and upper quartiles as the bottom and top edges of the boxes, respectively. The whiskers denote the data range, and the scatter points show individual values. The correlation between post- versus prestress reduction and improved mindfulness in parents is shown at the bottom right. Significant effects in parents survived multiple comparison false discovery rate (fdr) corrections (fdr corrected *P*<.05) for stress, anxiety, and depression. CDI: Child Depression Index. * represents that the *P* value is significant.

Notably, these patterns of behavioral change were mostly sustained at the 3-month follow-up after training completion (Figure 2). At follow-up, self-reported CDI scores were not significant relative to baseline (*z=*1.21; *d*=−0.24; 95% CI −11.43 to 3.54; *P*=.11); yet, parent-reported CDI scores showed a nonsignificant improvement in the expected direction (*z*=1.46; *d*=−0.25; 95% CI −5.57 to 0.90; *P*=.07). Parental self-reports at follow-up showed sustained stress reduction (*z*=1.72; *d*=−0.43; 95% CI −2.31 to 0.21; *P*=.05) as well as improvement in anxiety (*z*=1.87; *d*=−0.39; 95% CI −2.53 to 0.23; *P*=.03) and depression (PHQ-9, *z*=2.47; *d*=−0.81; 95% CI −4.46 to −0.68; *P*=.007) symptoms. Parents did not show a significant change in mindfulness (MAAS) at either postintervention or follow-up (*P*>.1); yet, notably, pre- versus posttraining reduction in parental stress correlated with improvement in mindfulness (ρ=−0.45; *P*=.03). We applied fdr corrections to account for multiple self-reported behavior comparisons in parents, and significant results did not change either for pre versus post or pre versus follow-up testing for parental stress, anxiety, and depression (*P*<.05 fdr-corrected).

### Cognitive Outcomes

**Figure 3.**
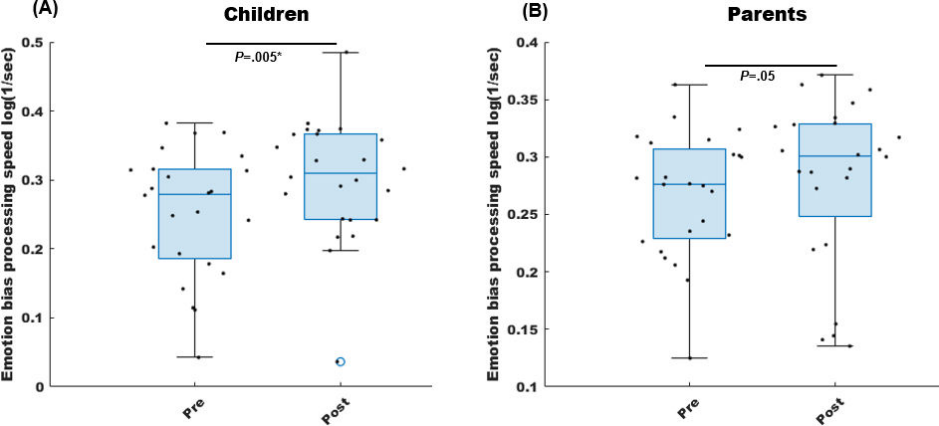
Swarm box charts of emotion bias task outcomes are shown for (A) children and (B) parents. Box plots show the median with the lower and upper quartiles as the bottom and top edges of the boxes, respectively. The whiskers denote the data range, and the scatter points show individual values. Processing speed units are log(1/sec), where sec is the seconds unit of average trial response time on the task. Greater processing speed corresponds to speedier (ie, smaller) response times or speedier cognitive task processing. * represents that the *P* value is significant.

Cognition was assessed as processing speed on the emotion bias task. Across all participants (n=48), there was a significant improvement in processing speed from pre- to postintervention (signed rank test *z*=3.42; *P*=.0006; *d*=0.40); yet, there was a significant group difference in this speed improvement (rank sum test *z*=2.01; *P*=.04). Post-hoc within-group pre- to postintervention signed rank tests revealed that only children showed a significant processing speed improvement across all emotion stimuli (*d*=0.54; 95% CI 0.012 to 0.083; *P*=.005), while adults showed a nonsignificant improvement in the expected direction (*d*=0.19; 95% CI −0.004 to 0.030; *P*=.05; Figure 3). In addition, children showed a consistent processing speed improvement for every emotion: neutral, happy, angry, or sad; that is, there was no bias toward a specific emotion (Table 3). The significant *P* values in children across emotion conditions survived fdr correction (*P*<.05).

**Table 3.** Processing speeds on the emotion bias task in children and adults.

Processing speed	Children			Parents		
Pre, mean (SD)^a^	Post, mean (SD)	*P* value	Pre, mean (SD)	Post, mean (SD)	*P* value
All faces	0.253 (0.09)	0.302 (0.09)	.005	0.268 (0.05)	0.280 (0.07)	.05
Neutral faces	0.255 (0.10)	0.307 (0.08)	.008	0.272 (0.06)	0.280 (0.07)	.21
Happy faces	0.249 (0.09)	0.306 (0.08)	.003	0.267 (0.05)	0.278 (0.07)	.09
Angry faces	0.258 (0.09)	0.301 (0.09)	.01	0.269 (0.05)	0.282 (0.08)	.14
Sad faces	0.250 (0.09)	0.296 (0.09)	.006	0.263 (0.06)	0.282 (0.07)	.05

a Mean and SD values are in log(1/sec), where sec is the seconds unit of average trial response time on the task. Post- versus precomparison *P* values are per signed rank tests.

### Neural Outcomes

**Figure 4.**
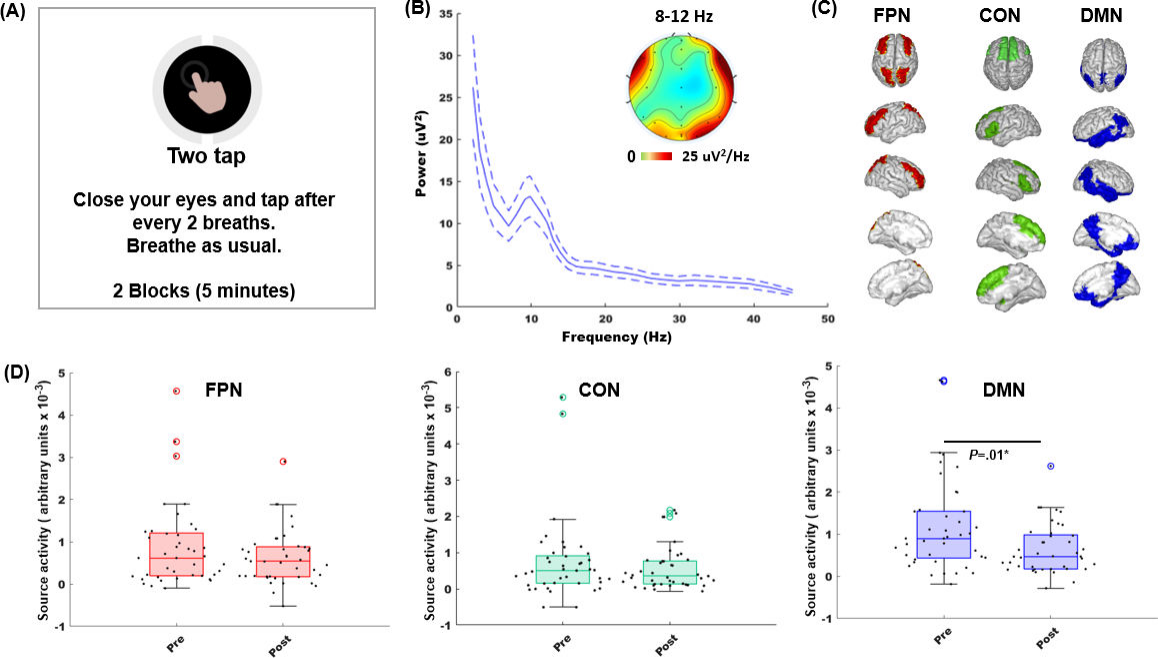
Cooperative Compassion training–related neurophysiological changes evaluated on the attention-to-breath monitoring assessment. (**A**) Schematic of attention-to-breath task instructions. (**B**) Power frequency plot of scalp channel data across all participants and sessions showed peak processing in the α frequency band (8‐12 Hz); dashed lines are 95% CIs. (**C**) Source-reconstructed electroencephalographic data were analyzed for 3 cognitive control networks: frontoparietal network (FPN), the cingulo-opercular network (CON), and the default mode network (DMN); regions of interest (ROIs) averaged within each network are highlighted. The identity of the ROIs within the 3 cognitive control networks is detailed in Multimedia Appendix 1. (**D**) Comparisons of the pre- to postintervention network changes across all participants are shown as swarm box plots. Box plots show median values with lower and upper quartiles as the bottom and top edges of the boxes, respectively. The whiskers denote the data range, and the scatter points show individual α source activity values.

Neural changes were evaluated in the 8‐12 Hz α frequency band of the EEG signal, as all participants showed peak processing in this band on the eyes-closed attention-to-breathing task. Across all participants, there was no significant change in FPN or CON source localized α activity (*P*>.4), but DMN activity was significantly reduced at postintervention relative to preintervention (signed rank test *z*=−2.48; *d*=−0.62; 95% CI −0.0096 to −0.0002; *P*=.01). In addition, there was no significant group difference in post- versus pre-DMN activity for children versus parents (rank sum test, *P*=.09; Figure 4). Group-specific post- versus prechanges in DMN activity showed significant reduction in DMN activity in children (signed rank test *z*=−2.56; *d*=−1.09; 95% CI −0.0015 to −0.0003; *P*=.01), but no change in parent (*P*>.5), suggesting that this neural outcome was exclusively driven by post- versus prechange in children.

The summary of post- versus prechanges in behavioral, cognitive, and neural outcome measures in parents and children is provided in Table 4.

**Table 4.** Summary of post- versus prechanges in behavioral, cognitive, and neural outcome measures in parents and children^a^.

Post- versus preintervention outcomes	Cohen *d* effect size	Mean difference (95% CI)	fdr^b^ corrected *P* value
Child depression (self-reported)	−0.19	−3.57 (−8.89 to 1.74)	ns^c^
Child depression (parent-reported)	−0.20	−1.83 (−4.93 to 1.27)	ns
Parental stress	−0.41	−1.39 (−2.63 to −0.16)	.03
Parental anxiety	−0.47	−1.44 (−2.6 to −0.20)	.03
Parental depression	−0.50	−1.67 (−3.25 to −0.08)	.03
Children emotion bias cognitive processing (all faces)	0.54	0.047 (0.012 to 0.083)	.01
Parent emotion bias cognitive processing (all faces)	0.19	0.013 (−0.004 to 0.030)	ns
DMN^d^ neural activity on attention-to-breath task (children and parents)	−0.62	−0.5631×10^−3^ (−0.0096 to −0.0002)	.04

a Children did not show any significant improvement in depressive symptoms, while parents showed significant improvement in stress, anxiety, and depression symptoms. Cognitively only children showed better emotion bias processing. Reduction in DMN neural activity was observed across all participants; there was no group interaction on this measure, but post-hoc tests showed this result to be driven by DMN neuroplasticity in children (see text).

b fdr: false discovery rate.

c ns: nonsignificant.

d DMN: default mode network.

## Discussion

The primary objective of our study was to develop a digital cotraining program that would facilitate parent and child copractice of breath-focused mindfulness and compassion. We found the CoCo digital trainings to be feasible with 80% (n=19) of families completing >90% (n=22) of sessions, and all families reporting positive feedback on an exit survey. We evaluated the pilot efficacy of this intervention program using behavioral surveys assessing depression in children by both self and parental reports as well as parental self-reported stress, anxiety, and depression symptoms. Overall, children showed a statistical trend toward, that is, nonsignificant improvement in the expected direction in self-reported depression at posttraining, while the parent-reported child depression symptoms showed a similar effect at 3-month follow-up. The parents showed significant reductions in their stress, anxiety, and depression symptoms at postintervention with sustained effects at the 3-month follow-up. Furthermore, the posttraining reduction in parental stress was significantly correlated with improvement in dispositional mindfulness. We further measured pre- versus posttraining cognition in the presence of emotional faces and found that emotion bias processing speed was significantly improved in children, and a nonsignificant effect in the expected direction was observed in parents. Finally, on a neural assessment of breath-focused attention, we found a significant reduction in DMN activity at postintervention relative to preintervention that was especially robust in children.

Our observation of training-related reduction in parental stress, anxiety, and depression symptoms resonates with previous meta-analyses of behavioral interventions showing positive mental health benefits in parents [25,48]. Notably, we found significant results with a brief digital intervention of only ~10 minutes per session practiced over 30 sessions in 3 months, which suggests that even brief interventions that families can practically engage with in their own time can be beneficial for parents. Statistically, we were powered for medium effect size outcomes (*d*>0.5) that are also clinically meaningful. These medium to large effect size outcomes were observed for parental depression (*d*=0.5 at post and *d*=0.8 at follow-up) but not for parental anxiety or stress. This suggests that the CoCo training may most robustly ameliorate parental depression. In terms of neurocognitive outcomes, the emotion bias processing speed improvements in children were robust with medium effect size results (*d*=0.54). This neurocognitive assessment is in line with recommendations that studies of parent-child interventions must include valid assessments of children’s emotion regulation abilities [22].

The neuroplasticity signal, specifically, reduction in DMN activity at postintervention relative to preintervention replicates our recent findings of digital mindfulness and compassion training–related neural changes observed in adults [44]. Specifically, this DMN activity modulation was observed on the attention-to-breath task, as the differential neural activity on high versus low consistency performance trials compared post- versus preintervention. As stated in the Methods section, the high versus low consistency trial contrast in neural activity controls for interindividual differences in baseline characteristics of respiration and reduction in DMN activity at postintervention relative to baseline may reflect reduced mind-wandering. This observation of intervention-related reduction in DMN activity also resonates with broader contemplative empirical research showing lesser DMN activity with greater mindfulness or meditation experience [49,50,52]. The nonsignificant effects within FPN and CON during the attention-to-breathing task may suggest the task-specific recruitment of DMN, as observed in our recent studies [44,45]. In this recent work, in a large sample of participants (n=324) across the lifespan, we showed that lesser DMN activity on the breath attention task relates to greater trait mindfulness [45]. Thus, the significant postintervention decrease in DMN activity, particularly in children, in our study may represent neuroplasticity reflective of reduced mind-wandering and greater present moment awareness and focus after the training [55-58]. It is also notable that we obtained these results using EEG, a more scalable approach for measuring neural markers compared to other more costly neuroimaging modalities like functional magnetic resonance imaging.

Major limitations of this pilot intervention study include a small sample size and no control intervention comparator. Given the small sample size, we can only be confident of the medium to large effect size results in this pilot study obtained for parental depression, cognitive processing speed on the emotion bias task in children, and DMN neuroplasticity analyzed across all participants. Another limitation of the study was the lack of inclusion of Black and multiracial parent-child dyads as well as families from low affluence socioeconomics. We also acknowledge that parent-reported CDI measures may have potential bias; hence, we considered both parent and self-reports on this measure. Additionally, the CoCo training included both mindfulness and compassion instructions to maximize benefit, and disentangling their respective contribution to the outcomes is not possible in this study. Future studies should certainly include an active digital training control (eg, a relaxation app) with a larger sample size and ensure that the sample is more representative of racial and SES diversity. Further, since the training program was designed to target compassion, a future trial should include a compassion outcome measure as well as a measure of caregiver-child relational interaction.

Overall, this study examined a promising, accessible, and scalable digital program for cotraining parents and children on aspects of mindfulness and compassion. Our results showed significant medium effect size improvements particularly for parental depression and smaller effect size improvements for parental stress and anxiety, as well as a nonsignificant reduction in the expected direction in depressive symptoms in children. Notably, these outcomes in parents were sustained at follow-up. Additionally, children showed significant medium effect size improvement in emotion bias–related cognitive processing and large effect size reduction of DMN activity on the attention-to-breathing task as a neural indicator of less mind-wandering or greater focus at posttraining. Compared to prior interventions, this study is novel in its use of dyadic parent-child cotraining design as well as multimodal outcome measures that combine EEG, cognitive, and behavioral data. Prior studies conducted separately in children and parents have particularly found improvements in mindfulness and stress-related behavioral measures of medium effect size, and one study showed improvements in stress-related cortisol levels in parents [79-82]. Mindfulness training in parents has also been shown to reduce externalizing psychopathologic behaviors in children [83], and such trainings in parents and children have been shown to reduce child ADHD symptoms [84-86]. No other studies to date in this literature have measured emotion-related cognitive processing or intervention-related DMN neuroplasticity. Future studies should certainly include an active digital training control (eg, a relaxation app that may also reduce stress) with a larger sample size and ensure that the sample is more representative of racial and SES diversity. Training program implementation in schools, clinical populations such as ADHD children, and underserved or trauma-exposed populations is also relevant. In conclusion, our encouraging findings support future scale-up of the CoCo program to nurture family well-being.

## Supplementary material

10.2196/69607 Multimedia Appendix 1 Additional material.

## Abbreviations

ADHD: attention-deficit/hyperactivity disorder
CDI: Child Depression Index
CoCo: Cooperative Compassion
CON: cingulo-opercular network
DASS-21: 21-item Depression Anxiety Stress Scale
DMN: default mode network
EEG: electroencephalographic
fdr: false discovery rate
FPN: frontoparietal network
GAD-7: 7-item General Anxiety Disorder
HIPAA: Health Insurance Portability and Accountability Act
MAAS: Mindful Attention Awareness Scale
PHQ-9: 9-item Patient Health Questionnaire
RT: response time
SES: socioeconomic status
