# Correction: Breath-Focused Mindfulness and Compassion Training in Parent-Child Dyads: Pilot Intervention Study

**DOI:** 10.2196/81122

**Published:** 2025-08-07

**Authors:** Satish Jaiswal, Jason Nan, Seth Dizon, Jessica O Young, Suzanna R Purpura, James K Manchanda, Dhakshin Ramanathan, Dennis J Kuo, Jyoti Mishra

**Affiliations:** 1 Department of Psychiatry, University of California, San Diego, 9500 Gilman Drive, Guava Building, Room 130, La Jolla, CA, 92093, United States, 1 6086092291; 2 VA San Diego Medical Center, San Diego, CA, United States

In “Breath-Focused Mindfulness and Compassion Training in Parent-Child Dyads: Pilot Intervention Study” (JMIR Form Res 2025;9:e69607), the authors noted one error.

In Table 1 of the originally published paper, the age ranges of parents and children were swapped. Table 1 with the age range corrected for “Parents (n=24)” and “Children (n=24)” is displayed below:

**Table 1.** Summary of demographics and baseline mental health for parent-child dyad study participants.^a^

Demographics and baseline mental health	Parents (n=24)	Children (n=24)
Age (years)		
Mean (SD)	44.5 (6.5)	9.5 (3.27)
Range	28-54	5-15

a Parental stress was measured using the 7 stress items on the 21-item Depression Anxiety Stress Scale (DASS-21), anxiety was measured on the 7-item General Anxiety Disorder (GAD-7) scale, and depression was measured on the 9-item Patient Health Questionnaire scale (PHQ-9).

The correction will appear in the online version of the paper on the JMIR Publications website, together with the publication of this correction notice. Because this was made after submission to PubMed, PubMed Central, and other full-text repositories, the corrected article has also been resubmitted to those repositories.

