# Correction: Breath-Focused Mindfulness and Compassion Training in Parent-Child Dyads: Pilot Intervention Study

**DOI:** 10.2196/89650

**Published:** 2026-03-02

**Authors:** Satish Jaiswal, Jason Nan, Seth Dizon, Jessica O Young, Suzanna R Purpura, James K Manchanda, Dhakshin Ramanathan, Dennis J Kuo, Jyoti Mishra

**Affiliations:** 1 Department of Psychiatry, University of California San Diego, 9500 Gilman Drive, Guava Building, Room 130, La Jolla, CA, 92093, United States, 1 6086092291; 2 VA San Diego Medical Center, San Diego, CA, United States

## Errors Identified

This corrigendum is being submitted for the article “Breath-Focused Mindfulness and Compassion Training in Parent-Child Dyads: Pilot Intervention Study” [1] in response to a PubPeer comment [2] regarding the neural data shared on Drayd. We found two major issues: (1) the duplications observed resulted from a batch processing script that erroneously plugged in repeat data, and (2) the subject IDs for pre- and postdata were misaligned when creating the final Dryad data Excel file.

## Addressing Errors

The following steps were taken to address the errors:

- We reprocessed all child neural data individually instead of as a batch process. In this iteration, all child neural data were successfully individually processed. Final data are now unique for each child participant, except child subject PJ24 (one of the subjects flagged in the PubPeer comment [2]), which could not be processed as they did not have any meaningful on-task responses (child continuously held down response spacebar throughout task), hence had to be excluded.
- As part of our processing pipeline involves across-subject outlier cleaning (as explained in Multimedia Appendix 1 of the original paper [1]), all data values are now updated (ie, values that were previously processed correctly are also slightly updated, with a 98% correlation between previous and current processing).
- We have doubly ensured that subject IDs and data are correctly aligned in the updated final data.

## Impact of the Errors on the Overall Findings

With the corrections made to the dataset, the overall conclusion of the study remains unchanged.

In the Neural Outcomes section, the statistics were changed from the following:

Across all participants, there was no significant change in FPN or CON source localized α activity (*P*>.4), but DMN activity was significantly reduced at postintervention relative to preintervention (signed rank test *z*=−2.48; *d*=−0.62; 95% CI −0.0096 to −0.0002; *P*=.01). In addition, there was no significant group difference in post- versus pre-DMN activity for children versus parents (rank sum test, *P*=.09; Figure 4). Group-specific post- versus prechanges in DMN activity showed significant reduction in DMN activity in children (signed rank test *z*=−2.56; *d*=−1.09; 95% CI −0.0015 to −0.0003; *P*=.01), but no change in parent (*P*>.5), suggesting that this neural outcome was exclusively driven by post- versus prechange in children.

This section now reads:

Across all participants, there was no significant change in FPN or CON source localized α activity (*P*>.1), but DMN activity was significantly reduced at postintervention relative to preintervention (signed rank test *z*=−3.21; *d*=−0.53; 95% CI −0.0010 to −0.0003; *P*=.001; Figure 4). There was a significant group difference in post- versus pre-DMN activity for children versus parents (rank sum test, *P*=.002). Group-specific post- versus prechanges in DMN activity showed significant reduction in DMN activity in children (signed rank test *z*=−3.46; *d*=−0.98; 95% CI −0.0015 to −0.0005; *P*=.0005), but no change in parent (*P*>.5), suggesting that this neural outcome was exclusively driven by post- versus prechange in children.

Additionally, these data updates are now reflected in the newly generated Figure 4D (attached here as Figure 1).

**Figure 1.**
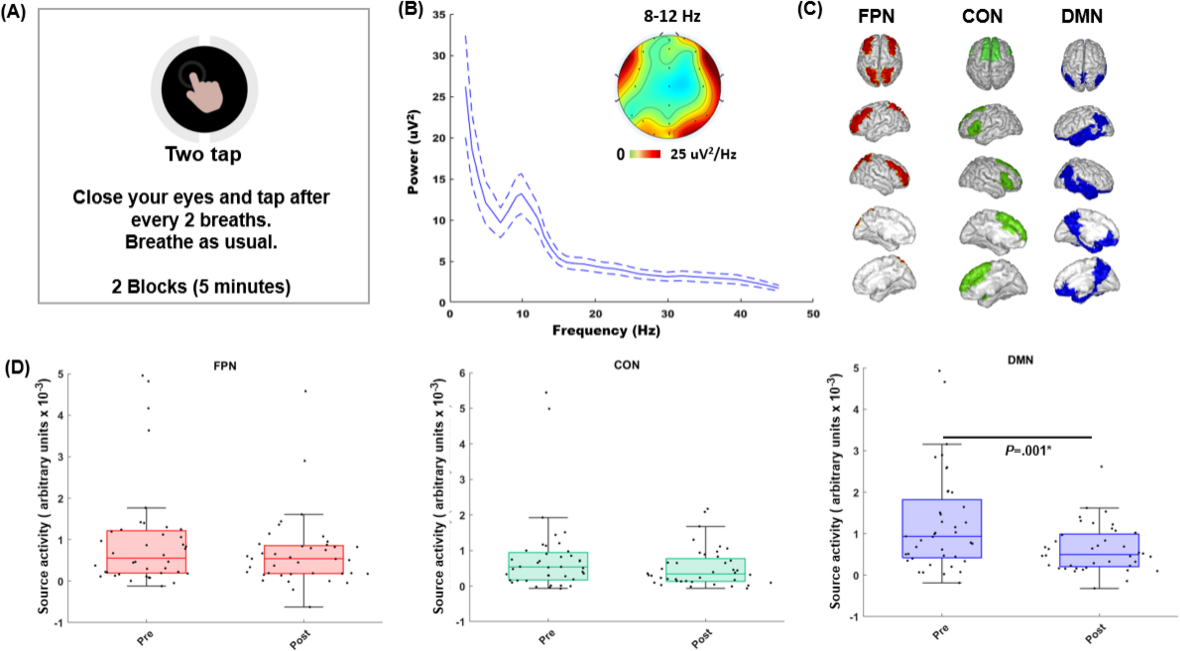
Cooperative compassion training–related neurophysiological changes evaluated on the attention-to-breath monitoring assessment. (A) Schematic of attention-to-breath task instructions. (B) Power frequency plot of scalp channel data across all participants and sessions showed peak processing in the α frequency band (8‐12 Hz); dashed lines are 95% CIs. (C) Source-reconstructed electroencephalographic data were analyzed for 3 cognitive control networks: frontoparietal network (FPN), the cingulo-opercular network (CON), and the default mode network (DMN); regions of interest (ROIs) averaged within each network are highlighted. The identity of the ROIs within the 3 cognitive control networks is detailed in Multimedia Appendix 1. (D) Comparisons of the pre- to postintervention network changes across all participants are shown as swarm box plots. Box plots show median values with lower and upper quartiles as the bottom and top edges of the boxes, respectively. The whiskers denote the data range, and the scatter points show individual α source activity values.

The last statement in the abstract was updated from the following:

Cortical source imaging of electroencephalographic recordings was acquired simultaneous to an attention-to-breathing assessment that showed significant reduction in task-related default mode network activity (*d*=−0.62; 95% CI −0.0096 to −0.0002; *P*=.01).

This sentence now reads:

Cortical source imaging of electroencephalographic recordings was acquired simultaneous to an attention-to-breathing assessment that showed significant reduction in task-related default mode network activity (*d*=−0.53; 95% CI −0.0010 to −0.0003; *P*=.001).

The false discovery rate–corrected statistics are shown in Table 4 (attached here as Table 1), with changed values shown in italics.

**Table 1.** Summary of post- versus prechanges in behavioral, cognitive, and neural outcome measures in parents and children^a^.

Post versus preintervention outcomes	Cohen *d* effect size	Mean difference (95% CI)	fdr^b^-corrected *P* value
Child depression (self-reported)	–0.19	–3.57 (–8.89 to 1.74)	ns^c^
Child depression (parent-reported)	–0.20	–1.83 (–4.93 to 1.27)	ns
Parental stress	–0.41	–1.39 (–2.63 to –0.16)	.03
Parental anxiety	–0.47	–1.44 (–2.6 to –0.20)	.03
Parental depression	–0.50	–1.67 (–3.25 to –0.08)	.03
Children emotion bias cognitive processing (all faces)	*0.54*	*0.049 (.011 to .088)*	*.01*
Parent emotion bias cognitive processing (all faces)	*0.19*	*0.013 (-.006 to .031)*	*ns*
DMN^d^ neural activity on attention-to-breath task (children and parents)	*–0.53*	-*0.0006 (-.0010 to -.0003)*	*.003*

a Children did not show any significant improvement in depressive symptoms, while parents showed significant improvement in stress, anxiety, and depression symptoms. Cognitively only children showed better emotion bias processing. Reduction in DMN neural activity was observed across all participants; there was no group interaction on this measure, but post-hoc tests showed this result to be driven by DMN neuroplasticity in children (see text).

b fdr: false discovery rate.

c ns: nonsignificant.

d DMN: default mode network.

The updated data files can be found on Dryad [3].

Lastly, we noted that in the previous corrected version, the correction in Table 1 regarding the age range of the children was only partially addressed. The age range for children was reported as:

5‐12

This now reads:

5-15

An updated table is shown below (attached here as Table 2), with changes marked in italics

The corrections will appear in the online version of the paper on the JMIR Publications website, together with the publication of this correction notice. Because these were made after submission to PubMed, PubMed Central, and other full-text repositories, the corrected article has also been resubmitted to those repositories.

**Table 2.** Summary of demographics and baseline mental health for parent-child dyad study participants^a^.

Demographics and baseline mental health	Parents (n=24)	Children (n=24)
Age (y)		
Mean (SD)	44.5 (6.5)	9.5 (3.27)
Range	28-54	*5-15*
Gender, n (%)		
Male	4 (16.7)	10 (41.7)
Female	20 (83.3)	14 (58.3)
Race, n (%)		
Asian	8 (33.3)	5 (20.8)
Black/African American	0 (0)	0 (0)
Native American	0 (0)	0 (0)
Native Hawaiian or Other Pacific	0 (0)	0 (0)
White	14 (58.3)	11 (45.8)
More than 1 ethnicity	2 (8.3)	7 (29.2)
Other	0 (0)	1 (4.2)
Ethnicity, n (%)		
Hispanic or Latino	5 (20.8)	5 (20.8)
Not Hispanic or Latino	18 (75)	19 (79.2)
Unknown	1 (4.2)	0 (0)
Socioeconomic status		
Mean (SD)	6.5 (1.4)	—^b^
Range	4-8	—
Child Depression Index *T* scores		
Mean (SD)	54.08 (9.06)	57.96 (13.75)
Range	40-90	37-74
Parental Stress (DASS-21)		
Mean (SD)	4.91 (3.61)	—
Range	0-13	—
Parental Anxiety (GAD-7)		
Mean (SD)	4.39 (3.28)	—
Range	0-14	—
Parental Depression (PHQ-9)		
Mean (SD)	5.13 (3.52)	—
Range	0-13	—

a Parental stress was measured using the 7 stress items on the 21-item Depression Anxiety Stress Scale (DASS-21), anxiety was measured on the 7-item General Anxiety Disorder (GAD-7) scale, and depression was measured on the 9-item Patient Health Questionnaire scale (PHQ-9).

b Not applicable.
