# Supplementary material for: Breath-Focused Mindfulness and Compassion Training in Parent-Child Dyads: Pilot Intervention Study
Source: JMIR Form Res. 2025 Jul 17;9:e69607. doi: 10.2196/69607 (PMC12289298; doi:10.2196/69607)
Supplement: Multimedia Appendix 1 [file formative-v9-e69607-s001.docx]

Supplementary Materials: Training Instructions and EEG Analysis Descriptions

**Compassion Training Instructions**

(shown in-app during daily introduction as text and audio, with each level presented for 6 training sessions)

- **Instructions Level 1**

**Settling the Mind**

Close your eyes and breathe naturally.

Tap after **each** **breath**. Keep the same pace throughout.

If your mind wanders and you forget to tap, or tap too fast or slow,

a gentle sound will remind you to bring your attention back to your breath.

Breathing Tip: Breathe through your nose.

Inhale & fill your belly with fresh air. Exhale with belly in.

**On Tablet: Tap | On Keyboard: Use Spacebar**

**Life Practice**: In your day, whenever you start to sense stress, pause and take a few deep breaths.

(shown on training completion page for level 1)

- **Instructions Level 2**

**Compassion for a Loved One**

Close your eyes and breathe naturally.

Tap after **every two** **breaths**. Keep the same pace throughout.

As you settle into the breathing practice, choose a friend or loved one.

Bring a favorite memory to mind.

Notice how you feel when you think of this loved one.

Notice any positive thoughts or feelings of gratitude when you think of this loved one.

Send your loved one silent caring wishes while your breathe.

“May you be happy. May you be free from suffering. May you be well.”

If your mind wanders and you forget to tap, or tap too fast or slow,

a gentle sound will remind you to bring your attention back to your breath.

Breathing Tip: Breathe through your nose.

Inhale & fill your belly with fresh air. Exhale with belly in.

**On Tablet: Tap | On Keyboard: Use Spacebar**

**Life Practice**: In your day, practice kindness on the go.

Offer wishes of loving-kindness to family and friends.

When loving-kindness shows up towards you, savor it.

Notice how it feels in your mind and body.

(shown on training completion page for level 2)

- **Instructions Level 3**

**Compassion for Oneself**

Close your eyes and breathe naturally.

Tap after **every three** **breaths**. Keep the same pace throughout.

As you settle into the breathing practice, first choose a friend or loved one and send them caring wishes.

“May you be happy.” “May you be free from suffering.” “May you be free from fear and anger.” “May you be well.”

Then create in your mind a loving compassionate image that represents wisdom, strength, acceptance, love, nurturing and caring. It could be a wise person you respect, a symbol, or an image from nature like the deep blue ocean, or the sun that shines light on all beings.

Imagine yourself in the presence of this compassion. Imagine that you can be totally relaxed and also draw on your own strength in the presence of this compassion. While you breathe, repeat silently -

“May I be happy. May I be free from suffering. May I be free from fear and anger. May I be well.”

If your mind wanders and you forget to tap, or tap too fast or slow,

a gentle sound will remind you to bring your attention back to your breath.

Breathing Tip: Breathe through your nose.

Inhale & fill your belly with fresh air. Exhale with belly in.

**On Tablet: Tap | On Keyboard: Use Spacebar**

**Life Practice**: In your day, notice any negative thoughts towards yourself.

Recognize these are just momentary thoughts and do not define you.

Replace negative thoughts with compassion towards yourself.

Magnify your positive thoughts and moments by bringing conscious awareness towards them.

(shown on training completion page for level 3)

- **Instructions Level 4**

**Loving-Kindness for Oneself**

Close your eyes and breathe naturally.

Tap after **every four** **breaths**. Keep the same pace throughout.

As you settle into the breathing practice, first choose a loved one and send them caring wishes.

“May you be happy.” “May you be free from suffering.” “May you be well.”

Then create in your mind a loving compassionate image. It could be a wise person you respect, a symbol, or an image from nature.

Imagine that you can be completely yourself and also draw on your own strength in the presence of this compassion. While you breathe, repeat silently -

“May I be happy. May I be free from suffering. May I be well.”

Also reflect: “In my heart of hearts, what is it that I truly aspire for? What do I want to offer to this world?”

Acknowledge the aspiration for genuine happiness and inner peace.

If your mind wanders and you forget to tap, or tap too fast or slow,

a gentle sound will remind you to bring your attention back to your breath.

Breathing Tip: Breathe through your nose.

Inhale & fill your belly with fresh air. Exhale with belly in.

**On Tablet: Tap | On Keyboard: Use Spacebar**

**Life Practice**: In your day, take a quiet moment to think of “What is truly meaningful to me in my life?”

*Without searching for the answer, just listen.*

Do something nice for yourself each day, however small.

Each evening list up to three things you feel grateful for.

Let the feelings of appreciation and gratitude permeate your mind body.

(shown on training completion page for level 4)

- **Instructions Level 5**

**Embracing Common Humanity**

Close your eyes and breathe naturally.

Tap after **every five breaths**. Keep the same pace throughout.

As you settle into the breathing practice, create in your mind a loving compassionate image.

Then picture a loved one and imagine that just like you, this person has had ups and downs in his or her life.

Just like you, this person has goals and dreams. Just like you, this person is of loving concern to someone.

Just like you, this person knows what pain, sadness, anger, fear feel like.

Just like you this person wants to love and be loved, to contribute and to be appreciated. Repeat silently:

“Just like me, this person wishes to be happy and free from suffering.”

Then bring to mind someone you recognize but don’t know well. For this person too, consider how they are just like you, feeling similar emotions and with similar aspirations. Repeat silently:

“Just like me, this person wishes to be happy and free from suffering.”

Feel the fundamental truth of this statement.

Many countless people play a supportive role in our lives, just as we play supportive roles in the lives of others, even those who we may never know. Take a moment to appreciate this interconnectedness. Let your heart and mind be touched by this feeling of common humanity that we share with others. Repeat silently:

“Just as I do, all others wish to be happy and free from suffering.”

If your mind wanders and you forget to tap, or tap too fast or slow,

a gentle sound will remind you to bring your attention back to your breath.

Breathing Tip: Breathe through your nose.

Inhale & fill your belly with fresh air. Exhale with belly in.

**On Tablet: Tap | On Keyboard: Use Spacebar**

**Life Practice**: In your day, practice compassion on the go.

Offer a compassionate wish to a loved one, to a friend, to an acquaintance, to a stranger to be happy, well, free from suffering, free from fear and anger, and at ease.

(shown on training completion page for level 5)

**Neuro-cognitive (EEG) Data Analyses.** This included: 1) EEG channel data processing, and 2) cortical source localization of the EEG data to estimate source-level neural activity.

1) EEG channel data processing was conducted using the EEGLAB toolbox v2020 in MATLAB v2020 EEG data was resampled at 250 Hz and filtered in the 1-45 Hz range to exclude ultraslow DC drifts at <1Hz and high-frequency noise produced by muscle movements and external electrical sources at >45Hz.

Within the attention to breathing task, EEG data were average-referenced and epoched to the LSL time-stamps of the response taps made by participants after every two breaths. Trials were epoched in the -4.0 sec to +4.0 sec window around response and categorized as either high consistency trials (trials with RT $\leq$ 1 median absolute deviation of median RT in each participant) or low consistency trials (trials with RT > 1 median absolute deviation of median RT in each participant) [1,2]. The [-4 +4] sec epoch was chosen because it was not contaminated by motor artifacts given that median response times for on-task two-breath monitoring across subjects were ~8 sec.

There were no missing channels in the EEG data across subjects. Epoched data were cleaned using the *autorej* function in EEGLAB to remove noisy trials, i.e. >5SD outliers rejected over max 8 iterations, followed by further cleaning of electrooculographic, electromyographic or non-brain source artifacts using the Sparse Bayesian learning (SBL) algorithm (https://github.com/aojeda/PEB). The cleaned data were then band filtered in the physiologically relevant theta (4-7 Hz), alpha (8-12 Hz), and beta (13-30 Hz) frequency bands. Given that alpha band oscillations are dominant during eyes closed state [3–6] as also evidenced in our data (see Results), we exclusively source localized alpha band neural processing.

2) We used the block-Sparse Bayesian learning (BSBL-2S) algorithm to localize the alpha frequency band filtered EEG data and partitioned the signals into cortical regions of interest (ROIs) and artifact sources. The BSBL-2S algorithm has been applied in several EEG studies[7–18]. BSBL-2S is a two-step algorithm in which the first-step is equivalent to low-resolution electromagnetic tomography (LORETA [19]. LORETA estimates sources subject to smoothness constraints, i.e., nearby sources tend to be co-activated, which may produce source estimates with a high number of false positives that are not biologically plausible. To guard against this, BSBL-2S applies sparsity constraints in the second step wherein blocks of irrelevant sources are pruned. Notably, this data-driven sparsity constraint reduces the effective number of sources considered at any given time as a solution, thereby reducing the uncertainty of the inverse solution. Thus, it is not that only higher channel density data can yield source solutions, the ill-posed inverse problem can also be solved by imposing more aggressive constraints on the solution to converge on the source model at lower channel densities, as also supported by prior research [20,21]. Of note, the BSBL-2S two-stage algorithm has been benchmarked to produce evidence-optimized inverse source models at 0.95AUC relative to the ground truth [22,23]. We have also shown that cortical source mapping with this method has high test-retest reliability (Cronbach’s alpha = 0.77, p<0.0001 [7] and have demonstrated that source ROI activations obtained from the 24-channel EEG have high correlations with those obtained with higher density 64-channel EEG (Spearman’s rho = 0.95±0.08 )[2].

For the source space localization in adults, ROIs were based on the standard 68 brain region Desikan-Killiany atlas [24] using the Colin-27 head model [25]. For children’s data, age specific head models were generated with the templates obtained from www.nitcr.org by the courtesy of Jer’s Lab (*Neurodevelopmental MRI Database - John E. Richards Lab*, 2024) [27,28]. Artifacts still remaining in source space within individual subject data were removed using the Grubbs statistical test applied iteratively using spline interpolation - an option available within the MATLAB isoutlier function and population outliers across all sessions and subjects source data were removed using the >5SD criterion. ROIs were further grouped into canonical cognitive control networks that can undergo task-dependent modulation, i.e. the fronto-parietal network (FPN), cingulo-opercular network (CON) and the default mode network (DMN) [29–32]. ROIs in each network are shown in Table S1 below.

| Network | Regions of Interest | ROI index in DK Atlas |
| --- | --- | --- |
| FPN | caudalmiddlefrontal L | 5 |
| FPN | caudalmiddlefrontal R | 6 |
| FPN | rostralmiddlefrontal L | 55 |
| FPN | rostralmiddlefrontal R | 56 |
| FPN | superiorparietal L | 59 |
| FPN | superiorparietal R | 60 |
| CON | caudalanteriorcingulate L | 3 |
| CON | caudalanteriorcingulate R | 4 |
| CON | insula L | 19 |
| CON | insula R | 20 |
| CON | parsopercularis L | 37 |
| CON | parsopercularis R | 38 |
| CON | parsorbitalis L | 39 |
| CON | parsorbitalis R | 40 |
| CON | parstriangularis L | 41 |
| CON | parstriangularis R | 42 |
| CON | superiorfrontal L | 57 |
| CON | superiorfrontal R | 58 |
| CON | transversetemporal L | 67 |
| CON | transversetemporal R | 68 |
| DMN (aDMN) | frontalpole L | 11 |
| DMN (aDMN) | frontalpole R | 12 |
| DMN (aDMN) | lateralorbitofrontal L | 25 |
| DMN (aDMN) | lateralorbitofrontal R | 26 |
| DMN (aDMN) | medialorbitofrontal L | 29 |
| DMN (aDMN) | medialorbitofrontal R | 30 |
| DMN (aDMN) | rostralanteriorcingulate L | 53 |
| DMN (aDMN) | rostralanteriorcingulate R | 54 |
| DMN (pDMN) | inferiorparietal L | 15 |
| DMN (pDMN) | inferiorparietal R | 16 |
| DMN (pDMN) | isthmuscingulate L | 21 |
| DMN (pDMN) | isthmuscingulate R | 22 |
| DMN (pDMN) | precuneus L | 51 |
| DMN (pDMN) | precuneus R | 52 |
| DMN (mtlDMN) | entorhinal L | 9 |
| DMN (mtlDMN) | entorhinal R | 10 |
| DMN (mtlDMN) | inferiortemporal L | 17 |
| DMN (mtlDMN) | inferiortemporal R | 18 |
| DMN (mtlDMN) | middletemporal L | 31 |
| DMN (mtlDMN) | middletemporal R | 32 |
| DMN (mtlDMN) | parahippocampal L | 35 |
| DMN (mtlDMN) | parahippocampal R | 36 |
| DMN (mtlDMN) | superiortemporal L | 61 |
| DMN (mtlDMN) | superiortemporal R | 62 |
| DMN (mtlDMN) | temporalpole L | 65 |
| DMN (mtlDMN) | temporalpole R | 66 |

**Table S1.** Regions of Interest (ROIs) grouped within the three cognitive control networks are enumerated for the fronto-parietal network (FPN), cingulo-opercular network (CON) and the default mode network (DMN). The DMN includes the anterior DMN (aDMN), posterior DMN (pDMN) and the medial temporal DMN (mtlDMN). Corresponding indices of ROIs within the Desikan-Killiany (DK) atlas are also shown. L: left, R: right.

**References:**

1. Jaiswal S, Purpura SR, Manchanda JK, et al. Design and Implementation of a Brief Digital Mindfulness and Compassion Training App for Health Care Professionals: Cluster Randomized Controlled Trial. *JMIR Ment Health*. 2024;11:e49467. doi:10.2196/49467

2. Ramanathan D, Nan J, Grennan G, et al. Modulation of posterior default mode network activity during interoceptive attention and relation to mindfulness. *Biol Psychiatry Glob Open Sci*. Published online August 23, 2024:100384. doi:10.1016/j.bpsgos.2024.100384

3. Barry RJ, Clarke AR, Johnstone SJ, Magee CA, Rushby JA. EEG differences between eyes-closed and eyes-open resting conditions. *Clin Neurophysiol*. 2007;118(12):2765-2773. doi:10.1016/j.clinph.2007.07.028

4. Kan DPX, Croarkin PE, Phang CK, Lee PF. EEG Differences Between Eyes-Closed and Eyes-Open Conditions at the Resting Stage for Euthymic Participants. *Neurophysiology*. 2017;49(6):432-440. doi:10.1007/s11062-018-9706-6

5. Li L. The Differences among Eyes-Closed, Eyes-Open and Attention States: An EEG Study. In: *2010 International Conference on Computational Intelligence and Software Engineering*. IEEE; 2010:1-4. doi:10.1109/WICOM.2010.5600726

6. Valipour S. Detection of an alpha rhythm of EEG signal based on EEGLAB. In: ; 2014. Accessed September 25, 2024. https://www.semanticscholar.org/paper/Detection-of-an-alpha-rhythm-of-EEG-signal-based-on-Valipour/ba48d60c070a7d93057b035e3f460f26f5fca894

7. Balasubramani PP, Ojeda A, Grennan G, et al. Mapping cognitive brain functions at scale. *NeuroImage*. 2021;231:117641. doi:10.1016/j.neuroimage.2020.117641

8. Balasubramani PP, Diaz-Delgado J, Grennan G, et al. Distinct neural activations correlate with maximization of reward magnitude versus frequency. *Cereb Cortex*. Published online December 26, 2022:bhac482. doi:10.1093/cercor/bhac482

9. Grennan G, Balasubramani PP, Vahidi N, Ramanathan D, Jeste DV, Mishra J. Dissociable neural mechanisms of cognition and well-being in youth versus healthy aging. *Psychol Aging*. 2022;37(7):827-842. doi:10.1037/pag0000710

10. Grennan G, Balasubramani PP, Alim F, et al. Cognitive and Neural Correlates of Loneliness and Wisdom during Emotional Bias. *Cereb Cortex*. 2021;31(7):3311-3322. doi:10.1093/cercor/bhab012

11. Fakhraei L, Francoeur M, Balasubramani PP, et al. Electrophysiological Correlates of Rodent Default-Mode Network Suppression Revealed by Large-Scale Local Field Potential Recordings. *Cereb Cortex Commun*. 2021;2(2):tgab034. doi:10.1093/texcom/tgab034

12. Shah RV, Grennan G, Zafar-Khan M, et al. Personalized machine learning of depressed mood using wearables. *Transl Psychiatry*. 2021;11(1):338. doi:10.1038/s41398-021-01445-0

13. Kato R, Balasubramani PP, Ramanathan D, Mishra J. Utility of Cognitive Neural Features for Predicting Mental Health Behaviors. *Sensors*. 2022;22(9):3116. doi:10.3390/s22093116

14. Nan J, Balasubramani PP, Ramanathan D, Mishra J. Neural dynamics during emotional video engagement relate to anxiety. *Front Hum Neurosci*. 2022;16:993606. doi:10.3389/fnhum.2022.993606

15. Ojeda J, Salomon AK, Rowe JK, Ban NC. Reciprocal Contributions between People and Nature: A Conceptual Intervention. *BioScience*. 2022;72(10):952-962. doi:10.1093/biosci/biac053

16. Nan J, Grennan G, Ravichandran S, Ramanathan D, Mishra J. Neural activity during inhibitory control predicts suicidal ideation with machine learning. *NPP—Digital Psychiatry Neurosci*. 2024;2(1):1-11. doi:10.1038/s44277-024-00012-x

17. Jaiswal S, Nan J, Purpura SR, et al. Resting state EEG source derived salience network theta connectivity mediates anxiety in community dwelling individuals reporting childhood trauma. *Int J Psychophysiol*. 2025;207:112486. doi:10.1016/j.ijpsycho.2024.112486

18. Nan J, Jaiswal S, Ramanathan D, Withers M, Mishra J. Climate Trauma from Wildfire Exposure Impacts Cognitive Decision-making. *Nat Sci Rep*. Published online 2025.

19. Pascual-Marqui RD, Michel CM, Lehmann D. Low resolution electromagnetic tomography: a new method for localizing electrical activity in the brain. *Int J Psychophysiol*. 1994;18(1):49-65. doi:10.1016/0167-8760(84)90014-X

20. Ding L, He B. Sparse source imaging in electroencephalography with accurate field modeling. *Hum Brain Mapp*. 2008;29(9):1053. doi:10.1002/HBM.20448

21. Stopczynski A, Stahlhut C, Larsen JE, Petersen MK, Hansen LK. The Smartphone Brain Scanner: A Portable Real-Time Neuroimaging System. *PLOS ONE*. 2014;9(2):e86733. doi:10.1371/JOURNAL.PONE.0086733

22. Ojeda A, Kreutz-Delgado K, Mullen T. Fast and robust Block-Sparse Bayesian learning for EEG source imaging. *NeuroImage*. 2018;174(July 2017):449-462. doi:10.1016/j.neuroimage.2018.03.048

23. Ojeda A, Kreutz-Delgado K, Mishra J. Bridging M/EEG Source Imaging and Independent Component Analysis Frameworks Using Biologically Inspired Sparsity Priors. *Neural Comput*. 2021;33:1-31. doi:10.1162/NECO_A_01415

24. Desikan RS, Ségonne F, Fischl B, et al. An automated labeling system for subdividing the human cerebral cortex on MRI scans into gyral based regions of interest. *NeuroImage*. 2006;31(3):968-980. doi:10.1016/j.neuroimage.2006.01.021

25. Holmes CJ, Hoge R, Collins L, Woods R, Toga AW, Evans AC. Enhancement of MR images using registration for signal averaging. *J Comput Assist Tomogr*. 1998;22(2):324-333. doi:10.1097/00004728-199803000-00032

26. Neurodevelopmental MRI Database - John E. Richards Lab. Accessed September 25, 2024. https://jerlab.sc.edu/projects/neurodevelopmental-mri-database/

27. Richards JE, Sanchez C, Phillips-Meek M, Xie W. A database of age-appropriate average MRI templates. *NeuroImage*. 2016;124(Pt B):1254-1259. doi:10.1016/j.neuroimage.2015.04.055

28. Richards JE, Xie W. Brains for all the ages: structural neurodevelopment in infants and children from a life-span perspective. *Adv Child Dev Behav*. 2015;48:1-52. doi:10.1016/bs.acdb.2014.11.001

29. Dosenbach NUF, Visscher KM, Palmer ED, et al. A Core System for the Implementation of Task Sets. *Neuron*. 2006;50(5):799-812. doi:10.1016/j.neuron.2006.04.031

30. Dosenbach NUF, Fair DA, Miezin FM, et al. Distinct brain networks for adaptive and stable task control in humans. *Proc Natl Acad Sci*. 2007;104(26):11073-11078. doi:10.1073/pnas.0704320104

31. Menon V, Uddin LQ. Saliency, switching, attention and control: a network model of insula function. *Brain Struct Funct*. Published online 2010:13.

32. Uddin LQ, Yeo BTT, Spreng RN. Towards a Universal Taxonomy of Macro-scale Functional Human Brain Networks. *Brain Topogr*. 2019;32(6):926-942. doi:10.1007/s10548-019-00744-6
